# Complete Genome Sequence of Klebsiella quasipneumoniae MMCC7, Isolated from an Eclectus Parrot (Eclectus roratus)

**DOI:** 10.1128/mra.00171-22

**Published:** 2022-04-25

**Authors:** M. M. C. Cheng, G. K. K. Lai, S. D. J. Griffin, F. C. C. Leung

**Affiliations:** a Shuyuan Molecular Biology Laboratory, The Independent Schools Foundation Academy, Hong Kong SAR, China; University of Maryland School of Medicine

## Abstract

Klebsiella quasipneumoniae MMCC7 is a multidrug- and heavy metal-resistant strain isolated from the feces of a pet shop eclectus parrot in Hong Kong. The complete genome, a single chromosome and circular plasmid (5,382,488 bp; G+C content, 57.79%), was determined by hybrid assembly.

## ANNOUNCEMENT

Within a genus that is very widely distributed (1–3), the Klebsiella pneumoniae complex (KPC), which incorporates the two subspecies of Klebsiella quasipneumoniae, is the most clinically relevant—including notable hospital-acquired infections during the COVID-19 pandemic (4–6). Multidrug resistance among these species is rapidly disseminated by mobile elements (4, 7), as is the hypervirulence of particular prevalence in the Asia-Pacific Rim (8–10). Although *K. quasipneumoniae* accounts for a minor proportion of clinical isolates worldwide, its incidence in avians and companion animals is common (11–13), and human infections are more frequently reported (14–16). In addition, while animals may be persistent reservoirs (17, 18), a broader role than zoonotic transfer is likely because KPC species are efficient vectors for antimicrobial resistance (AMR) gene transfer (4, 19).

MMCC7 was isolated from a fresh fecal sample from an 8-month-old eclectus parrot (Eclectus roratus) housed at a pet shop in Wanchai, Hong Kong. After serial dilution in phosphate-buffered saline, a 100-μL aliquot of the 0.01% (wt/vol) extract was spread onto CHROMagar orientation medium (BD Biosciences) and incubated overnight. A single selected metallic-blue colony was passaged 10 times on Luria agar and finally incubated overnight in Luria broth (all at 37°C) (20) before genomic DNA (gDNA) extraction (Invitrogen PureLink genomic DNA minikit).

Paired-end short-read sequencing libraries were prepared using the NexteraXT DNA library preparation kit and sequenced via the Illumina MiSeq platform using v3 chemistry (2 × 300 bp). Adapter sequences were removed using Trimmomatic v0.32 (21), and reads were quality-filtered and trimmed, producing 248,982 read pairs (mean length, 299 bp) totaling ~74.5 Mbp. Long-read libraries, prepared from the same extracted DNA using the rapid barcoding kit SQK-RBK004, were sequenced via Oxford Nanopore’s Spot-ON flow cell vR9, the MinION sequencer, and MinKNOW v3.1.8 software, with base-calling using Guppy v2.1.3. The final long-read data set, trimmed with Porechop v0.2.4 (22, 23), totaled 106,444 reads (757 Mbp) with a median length of 4,661 bp (*N*_50_, 13,618 bp). Default parameters were used for all software unless otherwise specified.

The complete genome sequence combined Illumina and MinION data sets using Unicycler v0.4.3 (24), yielding a circular chromosome of 5,187,804 bp (57.97% G+C content) and a circular plasmid of 194,684 bp (52.93% G+C content) with an average genome coverage of 37×, which were submitted to NCBI PGAP v5.0 (25) for annotation.

MMCC7 has an average nucleotide identity of 99.20% with type strain Klebsiella quasipneumoniae supbsp. *similipneumoniae* 07A044T (GenBank accession number CP084787) (26). It has the ST5013 sequence type, *wzi* allele 709, and chromosomal beta-lactamase OKP-B-6 (27), as determined using the Institute Pasteur multilocus sequence type (MLST) database (https://bigsdb.pasteur.fr/). Plasmid pMMCC7, classified as IncFIB(K)_14[CladeI] using the KpVR Web-based tool (https://bioinfo-mml.sjtu.edu.cn/KpVR/index.php) (28), contains heavy-metal resistance genes, including the *mer*RPTCA and *cop*ABCDRS operons (29, 30), and the *mrk*ABCDFJ operon coding type 3 fimbriae (31). It also encodes the AMR genes *blaTEM-1* (32), *aph(3″)-Ib*, *aph(6)-Id* (33), *tetD*, *macAB*, and *sul2*.

### Data availability.

The complete genome sequences and raw sequence data for *K. quasipneumoniae* MMCC7 are available through NCBI under BioProject number PRJNA759343, with GenBank accession numbers CP082785 (chromosome) and CP082786 (plasmid) and SRA numbers SRR15928120 (MinION) and SRR15928121 (MiSeq).
